# Effectiveness of an mHealth Program on Reducing Blood Pressure Among Young Adults With Prehypertension: Protocol of a Pragmatic Cluster Randomized Controlled Trial

**DOI:** 10.2196/67216

**Published:** 2025-08-07

**Authors:** Melita Sheilini, H Manjunatha Hande, Nagaraja Ravishankar, Akshay M J, Jyothi Nayak, Ramesh Chandrababu

**Affiliations:** 1 Department of Medical Surgical Nursing Manipal College of Nursing Manipal Academy of Higher Education Manipal India; 2 Kasturba Medical College, Manipal Manipal Academy of Higher Education Manipal India; 3 Vallabhbhai Patel Chest Institute University of Delhi New Delhi India; 4 KPMG London United Kingdom; 5 Sri Ramachandra Faculty of Nursing Sri Ramachandra Institute of Higher Education and Research Chennai India

**Keywords:** prehypertension, hypertension, blood pressure, cardiology, cardiac, young adult, randomized controlled trial, mobile-based program, global health, mHealth, mobile health, mobile app, smartphone, digital health, digital intervention

## Abstract

**Background:**

The prevalence of prehypertension may be underreported because of its asymptomatic nature. This study will use a pragmatic mobile health (mHealth) app to deliver the intervention, which is expected to be well received by the young adult population.

**Objective:**

This study aims to assess the prevalence of prehypertension among young adults and evaluate the effectiveness of an mHealth intervention in enhancing adherence to lifestyle practices, increasing knowledge on prehypertension and hypertension, and reducing blood pressure among participants in the experimental and control groups.

**Methods:**

The study consists of 2 phases. Phase 1 used a cross-sectional survey design, and phase 2 included a pragmatic randomized controlled trial with cluster randomization. The protocol was prepared according to the SPIRIT (Standard Protocol Items: Recommendations for Interventional Trials) 2013 checklist. The CONSORT (Consolidated Standards of Reporting Trials of Nonpharmacologic Interventions) 2017 guideline was followed for reporting the protocol. The estimated sample size for phase 1 was 762 participants, and the sample size for the phase 2 was 85 participants per group. Both the control and experimental groups will receive education on prehypertension and hypertension, with the experimental group additionally receiving an mHealth app. The experimental group’s adherence to lifestyle practices will be monitored every 15 days, and reminder messages will be sent to nonresponders. Both groups will be followed up with on-site after 1, 3, and 6 months to assess their knowledge on prehypertension and hypertension, blood pressure, and lifestyle practices. The primary outcome of the study will be an improvement in blood pressure readings. The secondary outcomes will include adherence to lifestyle practices and knowledge on prehypertension and hypertension.

**Results:**

This study was funded by the Indian Council of Medical Research (grant 2021-11792, received February 2023). The study is ongoing. Phase 1 was completed on March 29, 2024, with data collected from 762 samples from 8 randomly selected colleges from Udupi District. Currently, phase 2 is in progress. The data will be analyzed using jamovi software (version 2.3.21; The jamovi project). Phase 1 and 2 findings will be analyzed and the results will be published in a peer-reviewed journal. Ethical approval for the study was obtained in May 2022 (160/2022).

**Conclusions:**

Identifying high-risk groups of young adults with prehypertension enables early interventions to prevent future hypertension and combat the cardiovascular burden globally. The mHealth intervention is considered to be an effective strategy to impart knowledge and promote healthy behaviors among young adults. The study will contribute to achieving the United Nation’s Sustainable Development Goal 3, which focuses on ensuring good health and well-being.

**Trial Registration:**

Clinical Trial Registry of India CTRI/2023/04/051784; https://tinyurl.com/57y9enyd

**International Registered Report Identifier (IRRID):**

DERR1-10.2196/67216

## Introduction

Hypertension is the third most significant risk factor contributing to the burden of noncommunicable diseases (NCDs) and places a considerable public health burden on cardiovascular health in India [1]. According to the World Health Organization, hypertension is a leading cause of premature death, with prehypertension serving as a critical precursor to hypertension both nationally and globally [2]. The seventh report of the Joint National Committee classifies prehypertension as a systolic blood pressure of 120-139 mmHg or a diastolic blood pressure of 80-89 mmHg [3]. However, the eighth report of the Joint National Committee guidelines do not define prehypertension because their recommendations focus on individuals with diagnosed hypertension [4]. Despite the rising prevalence, increased awareness, and availability of treatments, hypertension remains poorly controlled, particularly in low-income and middle-income countries, leading to major cardiovascular events [2].

A study conducted in India revealed that the prevalence of hypertension and prehypertension among young adults is 11.2% and 33.3%, respectively [5]. Individuals with a family history of hypertension and those who are physically inactive have a higher risk of hypertension [6]. Identifying prehypertension during its asymptomatic stage allows for timely interventions to normalize blood pressure, thereby preventing or delaying the onset of hypertension [7].

Many young adults with increased blood pressure remain undiagnosed, and a substantial proportion of individuals are unaware of their existing prehypertension. Young people are one of India’s most valuable resources. Studies in India reported the prevalence of prehypertension among young adults to be in the range 24.6%-65% [5,7-13].

Mobile devices have become integral to health care, providing easy access to health information both at home and on the go [14]. Early identification of prehypertension enables proactive blood pressure management through preventive strategies, thereby reducing cardiovascular morbidity and mortality.

Globally, hypertension is the leading cause of death, accounting for 10.4 million deaths annually [15]. Prehypertension, the initial stage of hypertension, represents a critical point for intervention where appropriate measures can delay or prevent the progression to hypertension. In addition, prehypertension is associated with an increased risk of both hypertension and cardiovascular diseases (CVDs) [16].

A study conducted in the coastal villages of Udupi District in Southern India found that the prevalence of prehypertension among young adults (aged 20-30 years) was 45.2%. Biological factors, such as being in the 25- to 30-year age group, having preobesity or obesity, and behavioral factors, including sedentary occupations and excessive salt intake, were found to be associated with prehypertension [7].

Globally, CVDs account for approximately 17.9 million deaths among middle-aged adults, with hypertension being the most common cardiovascular disorder, responsible for 20%-50% of all CVD-related deaths. Once considered a condition mainly affecting adults, hypertension is now increasingly prevalent among younger populations, with its onset reported even in childhood [6,17].

Studies have reported a high prevalence of hypertension among Indian adults, with nearly 1 in 3 individuals having this condition. With approximately 762 million adults aged 18 years and older in India, this equates to an estimated 234 million adults with hypertension, indicating a significant future increase in the CVD burden. This finding highlights the urgent need for early detection and treatment because effective blood pressure control can prevent nearly one third of all cardiovascular-related deaths [15]. In addition to blood pressure management, therapeutic strategies should include modifying lifestyle, managing weight, and addressing other risk factors to reduce residual cardiovascular risk [18,19].

Health education within the community is vital to increase awareness about hypertension and its risk factors among younger generations. Promoting physical activity and healthy eating is critical to counter the rapid rise of NCDs in many low-income and middle-income countries. Leveraging modern information and communication technologies to deliver interventions for physical activity and dietary changes is particularly promising, given the increasing availability of these technologies in such regions [17].

The prevalence of prehypertension among adolescents is rising, though it remains underreported. This condition often progresses to hypertension in adulthood, with young adults experiencing cardiovascular and cerebrovascular disorders before the age of 40 or 45 years. This leads to significant morbidity and mortality and a substantial socioeconomic burden on society [6,20].

**Figure 1:**
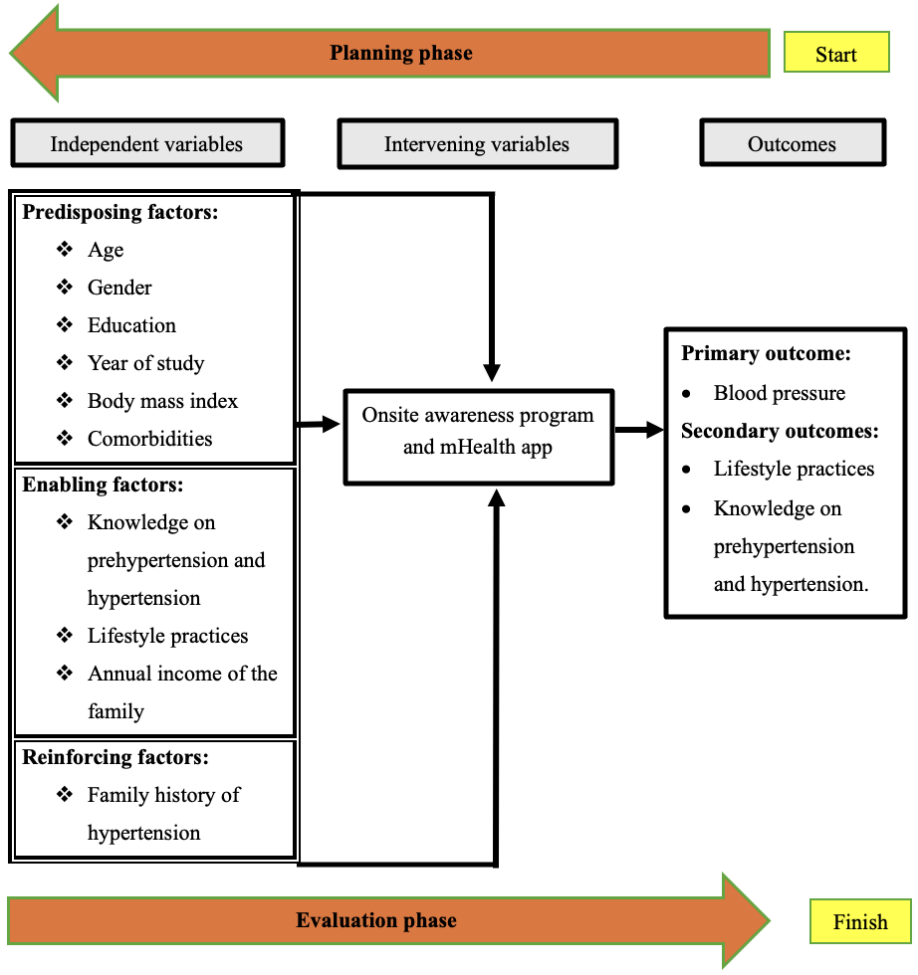
Conceptual framework on mHealth interventions for prehypertension among young adults based on the PRECEDE-PROCEED model developed by Green and Kreuter [23]. mHealth: mobile health; PRECEDE: predisposing, reinforcing, and enabling constructs in educational/environmental diagnosis and evaluation; PROCEED: policy, regulatory, and organizational constructs in educational and environmental development.

The conceptual framework adopted in this study is modified from the predisposing, reinforcing, and enabling constructs in educational/environmental diagnosis and evaluation (PRECEDE)-policy, regulatory, and organizational constructs in educational and environmental development (PROCEED) model [21,22] developed by Green and Kreuter [23] (Figure 1). Predisposing factors in the PRECEDE-PROCEED framework include antecedents to behavior, such as demographic and clinical factors, knowledge, and lifestyle behaviors. The enabling factors include existing knowledge on prehypertension and hypertension, lifestyle practices, and annual family income. The reinforcing factors include a family history of hypertension that encourages prompt health maintenance. The intervening variables are the on-site awareness program and the mobile health (mHealth) app aimed at enhancing knowledge on prehypertension and hypertension as well as adherence to healthy lifestyle practices. The outcome variables include blood pressure, knowledge on prehypertension and hypertension, and lifestyle practices.

Given the severity of this problem, the present study aims to determine the prevalence of prehypertension and evaluate the effect of mHealth interventions on adherence to lifestyle practices, knowledge on prehypertension and hypertension, and blood pressure among young adults. The growing adolescent population in India and the increasing risk factors highlight the importance of primary prevention as a crucial strategy to combat this epidemic.

This study aims to contribute to the United Nation’s Sustainable Development Goal 3 ensuring healthy lives and promoting well-being for all at all ages. The specific target is to reduce premature mortality from NCDs by one third through prevention and treatment and promote mental health and well-being by 2030.

## Methods

### Study Design

#### Overview

**Figure 2:**
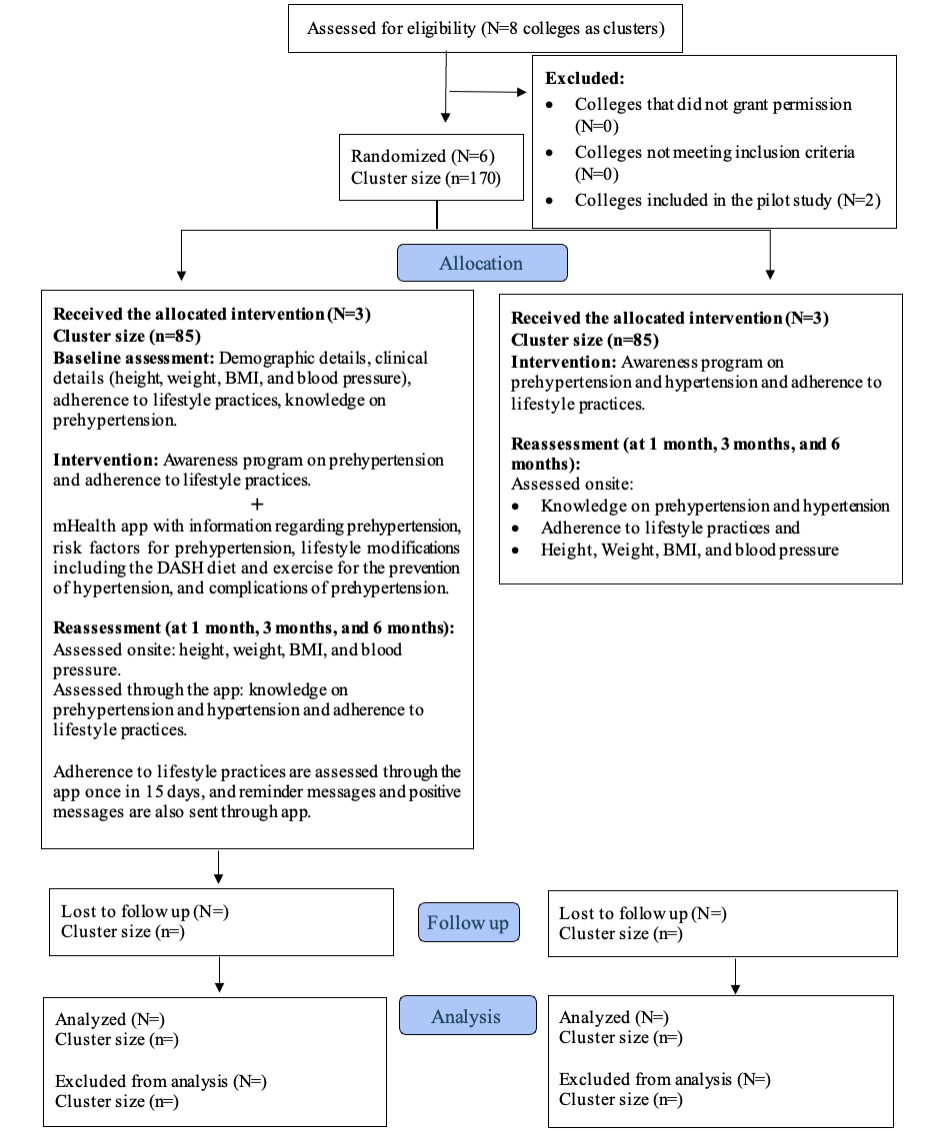
CONSORT (Consolidated Standards of Reporting Trials of Nonpharmacologic Interventions) 2017 flow diagram for the study. N denotes the number of colleges (ie, clusters) and n denotes the number of participants per cluster. DASH: dietary approach to stop hypertension.

The study is designed to be carried out in 2 phases. A cross-sectional survey design was used in phase 1. Because the study aims to evaluate the effect of the mHealth program on adherence to lifestyle practices, knowledge of prehypertension, and blood pressure, a pragmatic randomized controlled trial with cluster randomization was implemented in phase 2. The protocol follows the SPIRIT (Standard Protocol Items: Recommendations for Interventional Trials) 2013 checklist [24], which is provided in Multimedia Appendix 1. The CONSORT (Consolidated Standards of Reporting Trials of Nonpharmacologic Interventions) 2017 flowchart of the study is presented in Figure 2 [25-28].

#### Phase 1

After obtaining administrative permission from the Institutional Research Committee, Institutional Ethical Committee, and Joint Director of Collegiate Education, Udupi District, taluks from Udupi District were randomly selected to collect data for assessing the prevalence of prehypertension in phase 1. The data were collected on-site from young adults of colleges from randomly selected taluks. Baseline information, clinical variables, lifestyle practices, knowledge on prehypertension and hypertension, and blood pressure were measured for all participants from the selected colleges. An automated, digital, oscillometric blood pressure machine (Omron) was used to measure blood pressure after device calibration. As of March 29, 2024, phase 1 was completed with data collected from 762 samples from 8 randomly selected colleges.

#### Phase 2

Colleges with students having prehypertension identified during phase 1 were considered as clusters (N=6) and will be allocated to the intervention (N=3) and control groups (N=3) by simple randomization using the chit method. A random allocation sequence using the chit method was generated by research assistants who are not involved in the study. Currently, phase 2 is in progress.

### Intervention

#### Overview

The intervention protocol includes an on-site awareness program on prehypertension and hypertension (Table 1) and an mHealth app. The information in the mHealth app is provided under the following headings in both English and Kannada: about blood pressure, prehypertension and hypertension, diet, exercises, yoga, smoking and alcohol consumption, and sleep. The information leaflet encompassing the details of teaching provided during the on-site awareness program will be given to the participants of all the clusters after the study at the end of sixth month.

**Table 1:** Plan for the mHealth intervention. The total duration of teaching is 45 minutes.

Areas of teaching in the teaching plan	Duration (min)
Introduction	2
Meaning of prehypertension	2
Risk factors for prehypertension	5
Lifestyle modifications for the control of blood pressure	10
Significance of adherence to lifestyle practices in controlling blood pressure	5
Role of diet in reducing blood pressure focusing on DASH^a^ diet guidelines	5
Conclusion	1
Discussion	15

^a^DASH: dietary approach to stop hypertension.

#### mHealth App

**Figure 3:**
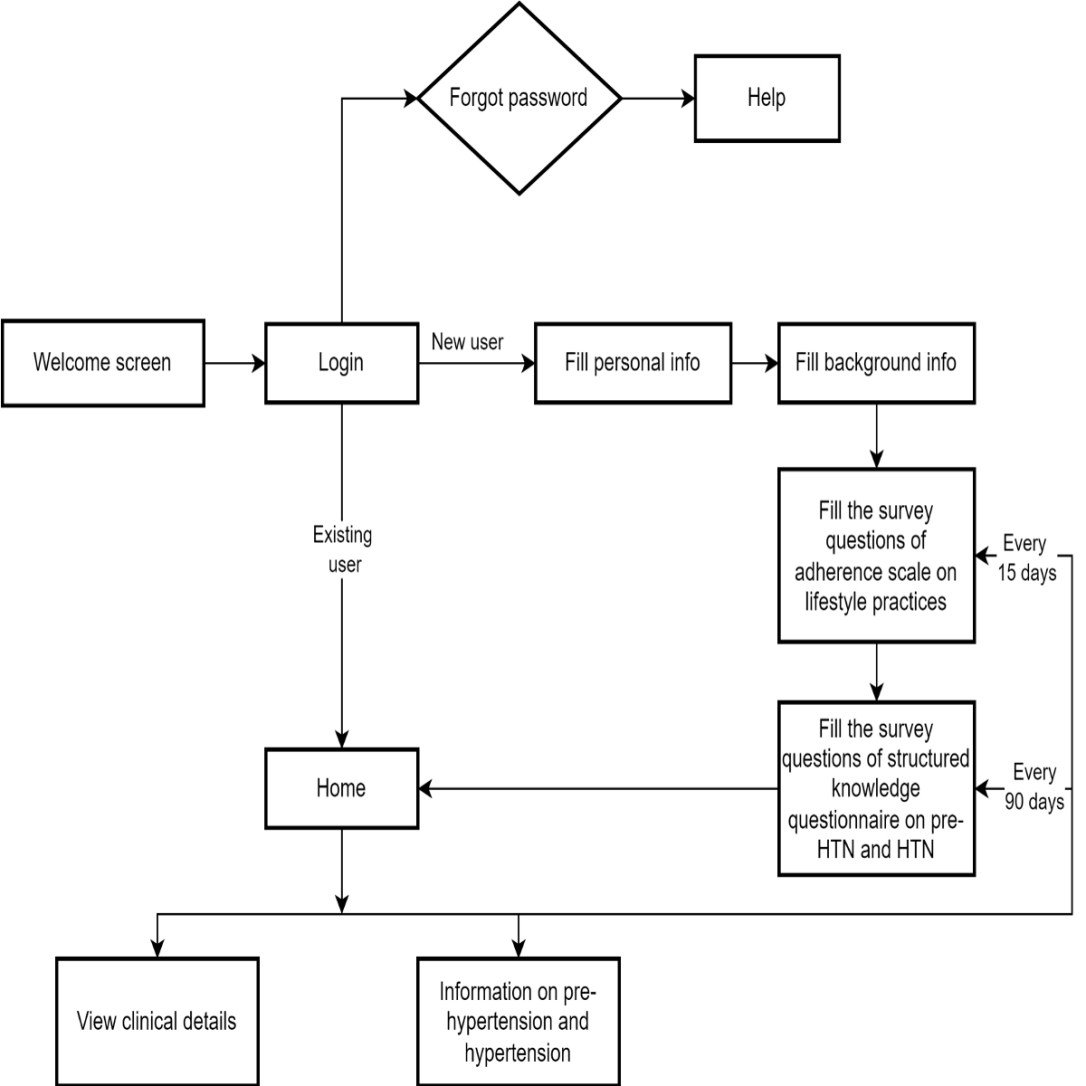
User flow diagram for the mHealth app on prehypertension. HTN: hypertension.

The mHealth app designed for the study, INFOBP, was customized to the research topic and the population of interest. The app was designed with the support of the team from Information Technology. It provides information on prehypertension and its risk factors as well as lifestyle modifications, including the dietary approach to stop hypertension (DASH) diet and exercise, for preventing and managing the complications of prehypertension. The information provided in the app is available in both English and Kannada. When responding to questionnaires, the participants have the freedom to make changes to their responses until they submit the questionnaire. Once submitted, no changes can be made from the user’s end. The information provided in the app can be viewed by the participants any number of times at their own pace. The app’s user flow is depicted in Figure 3.

A screenshot of the mHealth app is presented in Multimedia Appendix 2. To evaluate the validity and applicability of eHealth trials, the CONSORT-EHEALTH (Consolidated Standards of Reporting Trials of Electronic and Mobile Health Applications and Online Telehealth) checklist [29] was used and is provided in Multimedia Appendix 3. To check the quality and usefulness of the mHealth app, the mobile application rating scale (Multimedia Appendix 4) [30,31] was used by 12 participants from the experimental group after a few days of app usage. The participants’ responses are provided in Multimedia Appendix 5. Due permission was obtained from the original author for using the mobile application rating scale.

Both the control and intervention groups will receive education on prehypertension. The experimental group will also receive the mHealth app free of cost, provides information on prehypertension, risk factors for prehypertension, lifestyle modifications (including the DASH diet and exercise), and strategies for preventing hypertension and its complications. The mHealth app (INFOBP) will be installed on the mobile devices of all students in the intervention group, and they will receive orientation on its use. The experimental group will be monitored for adherence to lifestyle practices every 15 days through the mobile app, and reinforcement and reminder messages will be sent to nonresponders. All participants in the experimental group will receive a message of encouragement through the app every 15 days to continue practicing the recommended lifestyle changes. The participants will also be able to view the comparative report of their clinical parameters (BMI and blood pressure) and the result of the data entered by them (knowledge and lifestyle adherence scores) during every assessment as feedback. Both groups will be followed up after 1, 3, and 6 months to assess their knowledge on prehypertension and adherence to lifestyle practices and record their height, weight, BMI, and blood pressure on-site. At the end of the study, an information booklet on prehypertension will be provided to every college in Udupi District that was included in the study.

#### Study Participants and Sampling

The study will be conducted in the Udupi District of Karnataka, which is divided into 7 taluks (Udupi, Kundapur, Karkala, Hebri, Byndoor, Brahmavar, and Kapu). There are 33 private and 10 government-run colleges in the district. Young adults aged 18-25 years working toward a bachelor of science, bachelor of communication, or bachelor of arts in colleges in Udupi District who meet the inclusion and exclusion criteria (Textbox 1) will be considered for the study.

**Inclusion and exclusion criteria for the study.**

**Phase 1**

- Inclusion criteria
  Young adults aged between 18-25 years studying in selected degree colleges in Udupi District, Karnataka.
  Young adults willing to participate in the study.
- Exclusion criteria
  Young adults who have already been diagnosed with hypertension or other systemic disorders.
  Young adults who are on medications for any other ailments.

**Phase 2**

- Inclusion criteria
  Young adults identified to have prehypertension in phase 1.
  Young adults with prehypertension willing to participate in the study.
  Young adults with prehypertension having android mobile phones.
  Colleges with students having prehypertension identified during phase 1 were considered.
- Exclusion criteria
  Young adults identified to have hypertension in phase 1 and who were already diagnosed with other systemic disorders.
  Young adults with prehypertension who are on medications for any other ailments.
  Young adults with prehypertension who do not have android mobile phones.

The calculated sample size for phase 1 was determined to be 381 participants using the following formula for estimating a proportion, where n is the sample size required, Z_1-α/2_ is the desired confidence level (ie, for a 95% confidence level, Z_1-α/2_=1.96), P is the prevalence, and d is the margin of error:

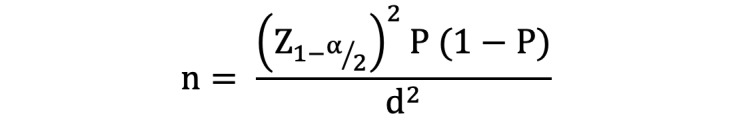



This calculation assumes the prevalence of prehypertension among young adults to be 45% at a 95% confidence level with a 5% margin of error (absolute precision) based on a previous study by Kini et al [7]. After multiplying by a design effect of 2 due to cluster sampling, the sample size was determined to be 762 participants.

The sample size for phase 2 was 34 participants per group, considering a 38% anticipated population SD of the primary outcome variable (ie, the prevalence of prehypertension) obtained using the equation for repeated measures ANOVA given below. In the equation, n is the sample size required, Z_1-α/2_ is the desired confidence level (ie, for a 95% confidence level, Z_1-α/2_=1.96), Z_1-β_ is the power of the test (ie, for a power of 80%, Z_1-β_=0.84), σ² is the population variance (ie, square of the SD), m is the number of groups being compared, ρ is the correlation coefficient between groups, and d is the minimum detectable difference.

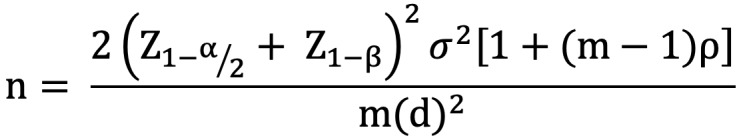



Accounting for a 20% dropout rate, the final sample size was 43 participants per group. After multiplying by a design effect of 2 due to cluster randomization, the sample size was determined to be 85 participants per group.

### Outcomes

The primary outcome is blood pressure, and the secondary outcomes are adherence to lifestyle practices and knowledge on prehypertension and hypertension.

### Data Collection Procedure and Instruments

The baseline data will be collected using a tool with 2 sections: demographic and clinical parameters. Adherence to lifestyle practices will be assessed using the adherence scale on lifestyle practices developed by the researchers, with a maximum score of 12. Adherence will be categorized as “adherent” for those achieving ≥80% (a score of ≥10) and “nonadherent” for those scoring <80% (a score of <10). A structured knowledge questionnaire on prehypertension and hypertension will be used to assess knowledge on the subject. The questionnaire consists of 30 items, with a maximum score of 30. The items are based on the Joint National Committee 7 recommendations for nonpharmacological interventions in the management of prehypertension. Knowledge levels will be classified as poor (a score of 0-9), average (a score of 10-20), or good (a score of 21-30). Blood pressure will be assessed using a digital blood pressure apparatus. The baseline blood pressure will be measured after a 5-minute to 10-minute rest period. Two readings will be taken 1-3 minutes apart, and the average will be calculated. In case of a difference of more than 5 mmHg between the 2 readings, an additional 2 readings will be obtained, and the average of these multiple readings will be used [32]. Prehypertension will be identified among young adults based on the average of 2 blood pressure readings. The same procedure will be followed during the follow-up sessions at 1, 3, and 6 months. A calibrated stadiometer and weighing machine will be used to assess the heights and weights of the participants.

The validated tools were tested for reliability on 20 students from the selected degree colleges in Udupi District. The reliability of the structured knowledge questionnaire was assessed using the split-half method (*r*=0.911). The tools were pretested with 10 students from a degree college in Udupi District. A pilot study was conducted with 20 students at a selected degree college.

### Ethical Considerations

The study protocol received institutional ethical approval on May 11, 2022 (IEC1 160/2022) and is registered under the Clinical Trial Registry of India (CTRI/2023/04/051784). All the participants will receive a subject information sheet in both Kannada and English to help them understand the study. Written informed consent will be obtained from all participants prior to data collection. The study will adhere to the guidelines of the Declaration of Helsinki. The confidentiality and anonymity of the participants will be maintained.

### Statistical Analysis

Data analysis will be conducted using jamovi (version 2.3.21; The jamovi project), using both descriptive and inferential statistics in line with the study objectives. For descriptive statistics, means and SDs will be used because all study outcomes are quantitative and continuous. Because the study involves assessing outcomes at 4 time points (baseline, 1 month, 3 months, and 6 months), repeated measures ANOVA will be used to evaluate the effectiveness of the intervention, and per protocol analysis will be used.

## Results

Ethical approval was obtained on May 11, 2022 (IEC1 160/2022), and the Indian Council of Medical Research provided funding for this project on February 9, 2023 (Project ID:2021-11792). The project is still in progress. On March 29, 2024, phase 1 was completed, with data gathered from 762 samples from 8 randomly chosen colleges in the Udupi District. Phase 2 is now underway (Table 2). The outcomes of phase 1 and phase 2 will be published in reputable peer-reviewed journals.

**Table 2:** Progress of phase 2 detailing the timelines of sample assessment.

Time	Baseline	1 month	3 months	6 months
Intervention group	September 16 to November 19, 2024	October 16 to December 20, 2024	January 2 to February 28, 2025	Started on April 4, 2025, and is expected to be completed by June 30, 2025
Control group	September 19 to October 19, 2024	October 30 to November 29, 2024	January 2 to February 28, 2025	Started on April 4, 2025, and is expected to be completed by June 30, 2025

## Discussion

### Study Overview

The primary objective of the study is to enhance awareness on prehypertension and hypertension among young adults, the productive population of the country, thereby promoting positive health and preventing premature CVDs. Globally, hypertension is responsible for 10.4 million deaths annually [15]. Prehypertension, an early stage of hypertension, can be effectively managed with proactive measures, thereby delaying or preventing the future risk of hypertension and its complications [16].

Evidence from a study conducted in Southern India suggests a 45.2% prevalence of prehypertension among young adults (aged 20-30 years). The study also revealed that prehypertension risk is associated with obesity and several lifestyle factors, including unhealthy dietary habits and occupation-related physical inactivity [7]. Although hypertension was once considered a disease of adults, studies now show an alarming rise in cases among younger age groups [2].

CVD accounts for approximately 17.9 million deaths globally among middle-aged adults, with 20%-50% of these deaths attributed to hypertension-related cardiovascular conditions [2]. In India, hypertension is highly prevalent, affecting 1 in 3 adults. With an estimated 762 million people aged 18 years or older in India, this translates to 234 million adults living with hypertension. The rising prevalence of hypertension, particularly among young adults, poses a significant socioeconomic burden if not addressed promptly [9,18]. Globally, the number of adults with hypertension, estimated at 1 billion in 2000, is expected to double to 2 billion by 2025 [33]. Research strongly supports the role of lifestyle interventions as an essential measure in effective blood pressure control and the reduction of CVD-related morbidity and mortality [9,17,20].

Smartphones are becoming a part of almost everyone’s lives, serving as essential tools for work, leisure, and communication. Smartphones are changing the way health education is delivered, promoting wellness activities and easing the management of diseases across a wide spectrum of populations [34]. The anticipated success of the mHealth app used in this study can be attributed to its customized approach. The app was developed as a blended approach to assess knowledge and lifestyle practices, track clinical parameters of participants with prehypertension, and disseminate information pertaining to health promotion. Any positive outcomes observed in this study would suggest that the mHealth app can be successfully used, supporting its extension to the population at large.

This protocol addresses a burning issue, and the methodology emphasizes assessing the real-world impact of the mHealth app. The effectiveness of digital health interventions in managing hypertension has led to successful blood pressure control. This approach could also be explored for its potential in weight management and obesity prevention. By supporting healthy weight maintenance and encouraging the development of healthy eating habits, particularly among students, these interventions may contribute to improved heart health and, more significantly, help prevent hypertension and CVDs [35-37]. Another study found that a youth-led digital intervention was effective in controlling blood pressure among adults with hypertension [38]. The growing population in India, coupled with increasing risk factors, supports the importance of primary prevention as a critical strategy to combat this epidemic. Based on the relevant research and existing data, this protocol underlines the importance of mobile health interventions in addressing prehypertension among young adults in this digital era.

### Limitations

A limitation of this study is that it is confined to young adults studying at colleges in Udupi District, Karnataka, which restricts the generalizability of the findings. Additionally, the data collected to assess knowledge and lifestyle practices are self-reported by the participants.

### Strengths

The cluster randomized controlled trial and customized app developed as part of the intervention are strengths of this study. Furthermore, research assistants are collecting the data and the researchers are not directly contacting the study participants, thus blinding the researchers.

### Conclusions

Prehypertension is associated with a higher likelihood of developing hypertension and doubles the risk of cardiovascular events. Given the severity of this issue, this study is timely. In this study, mHealth interventions were designed to target individuals identified as having prehypertension. Further research is recommended to develop interventions that can detect early signs of CVDs in individuals with prehypertension. Because young adults represent the nation’s most valuable resource and contribute significantly to its growth, prioritizing innovative interventions to combat prehypertension is essential for building a healthier nation.

## Appendix

Multimedia Appendix 1 SPIRIT 2013 checklist.

Multimedia Appendix 2 Screenshot of the mobile health app.

Multimedia Appendix 3 CONSORT-eHEALTH checklist (V 1.6.1).

Multimedia Appendix 4 Mobile application rating scale (MARS).

Multimedia Appendix 5 Response of the participants on the INFOBP app obtained using the mobile application rating scale.

## Abbreviations

CONSORT: Consolidated Standards of Reporting Trials of Nonpharmacologic Interventions
CONSORT-EHEALTH: Consolidated Standards of Reporting Trials of Electronic and Mobile Health Applications and Online Telehealth
CVD: cardiovascular disease
DASH: dietary approach to stop hypertension
mHealth: mobile health
NCD: noncommunicable disease
PRECEDE: predisposing, reinforcing, and enabling constructs in educational/environmental diagnosis and evaluation
PROCEED: policy, regulatory, and organizational constructs in educational and environmental development
SPIRIT: Standard Protocol Items: Recommendations for Interventional Trials

## Data Availability

Supporting data cannot be made publicly available due to ethical issues and its proprietary nature. The data may be accessed upon reasonable request to the corresponding author and following the publication of the study results.
