# Supplementary material for: Effectiveness of an mHealth Program on Reducing Blood Pressure Among Young Adults With Prehypertension: Protocol of a Pragmatic Cluster Randomized Controlled Trial
Source: JMIR Res Protoc. 2025 Aug 7;14:e67216. doi: 10.2196/67216 (PMC12371297; doi:10.2196/67216)
Supplement: Multimedia Appendix 1 [file resprot_v14i1e67216_app1.docx]

| **Time Point** | **Enrollment** | **Allocation** | **Post allocation** | | | | |
| --- | --- | --- | --- | --- | --- | --- | --- |
|  |  |  | Baseline | Once in 15 days | 1^st^ month | 3^rd^ month | 6^th^ month |
| **Enrollment** |  |  |  |  |  |  |  |
| Eligibility screen | x |  |  |  |  |  |  |
| Informed consent | x |  |  |  |  |  |  |
| **Allocation** |  | x |  |  |  |  |  |
| **Interventions** |  |  |  |  |  |  |  |
| mHealth program on blood pressure |  |  | x |  |  |  |  |
| Awareness program on prehypertension and adherence to lifestyle practices |  |  | x |  |  |  |  |
| **Assessments** |  |  |  |  |  |  |  |
| Demographic details | x |  | x |  |  |  |  |
| Clinical details (Height, Weight, BMI, Blood Pressure) |  |  | x |  | x | x | x |
| Knowledge of Prehypertension and Hypertension |  |  | x |  | x | x | x |
| Adherence to lifestyle practices |  |  | x | x | x | x | x |

Table 3. Standard Protocol Items: Recommendations for Interventional Trials (SPIRIT)
