# Supplementary material for: Effectiveness of an mHealth Program on Reducing Blood Pressure Among Young Adults With Prehypertension: Protocol of a Pragmatic Cluster Randomized Controlled Trial
Source: JMIR Res Protoc. 2025 Aug 7;14:e67216. doi: 10.2196/67216 (PMC12371297; doi:10.2196/67216)

INFOBP

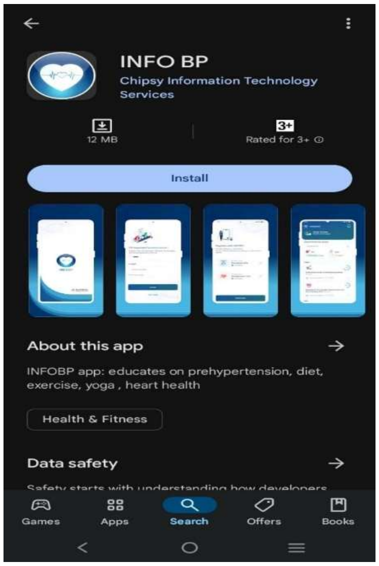
INFO BP
Chipsy Information Technology Services
12 MB
Rated for 3+
Install
About this app
INFOBP app: educates on prehypertension, diet, exercise, yoga , heart health
Health & Fitness
Data safety
Games
Apps
Search
Offers
Books

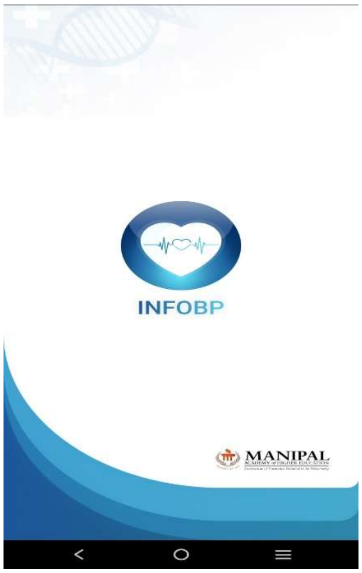
INFOBP
MANIPAL

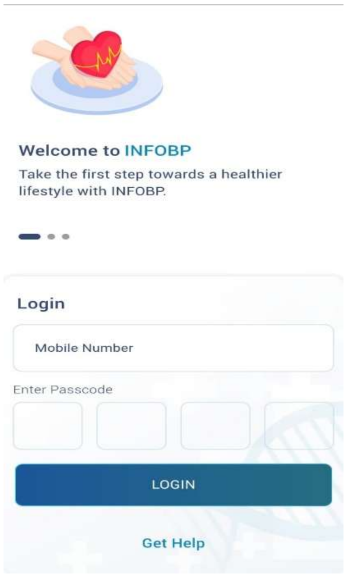
Welcome to INFOBP
Take the first step towards a healthier lifestyle with INFOBP.
Login
Mobile Number
Enter Passcode
LOGIN
Get Help

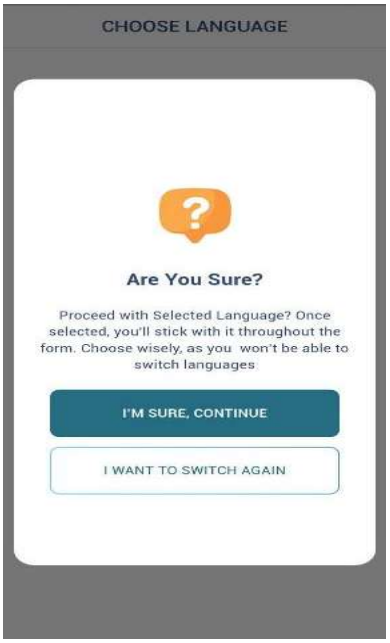
CHOOSE LANGUAGE
Are You Sure?
Proceed with Selected Language? Once selected, you'll stick with it throughout the form. Choose wisely, as you won't be able to switch languages
I'M SURE, CONTINUE
I WANT TO SWITCH AGAIN

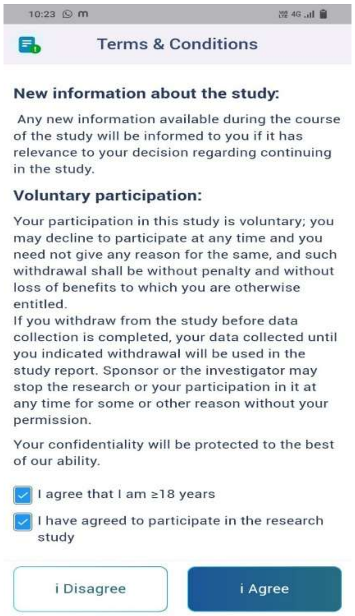
10:23
Terms & Conditions
New information about the study:
Any new information available during the course of the study will be informed to you if it has relevance to your decision regarding continuing in the study.
Voluntary participation:
Your participation in this study is voluntary; you may decline to participate at any time and you need not give any reason for the same, and such withdrawal shall be without penalty and without loss of benefits to which you are otherwise entitled.
If you withdraw from the study before data collection is completed, your data collected until you indicated withdrawal will be used in the study report. Sponsor or the investigator may stop the research or your participation in it at any time for some or other reason without your permission.
Your confidentiality will be protected to the best of our ability.
I agree that I am ≥18 years
I have agreed to participate in the research study
i Disagree
i Agree

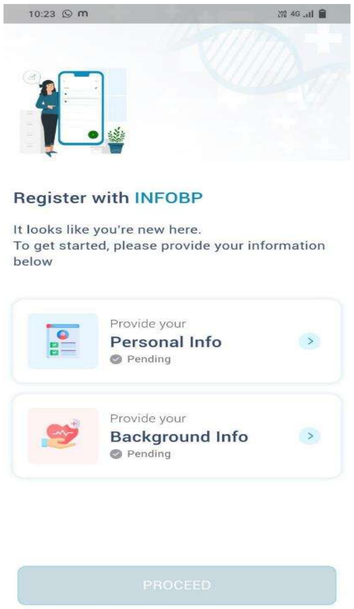
10:23
Register with INFOBP
It looks like you're new here.
To get started, please provide your information below
Provide your
Personal Info
Pending
Provide your
Background Info
Pending
PROCEED

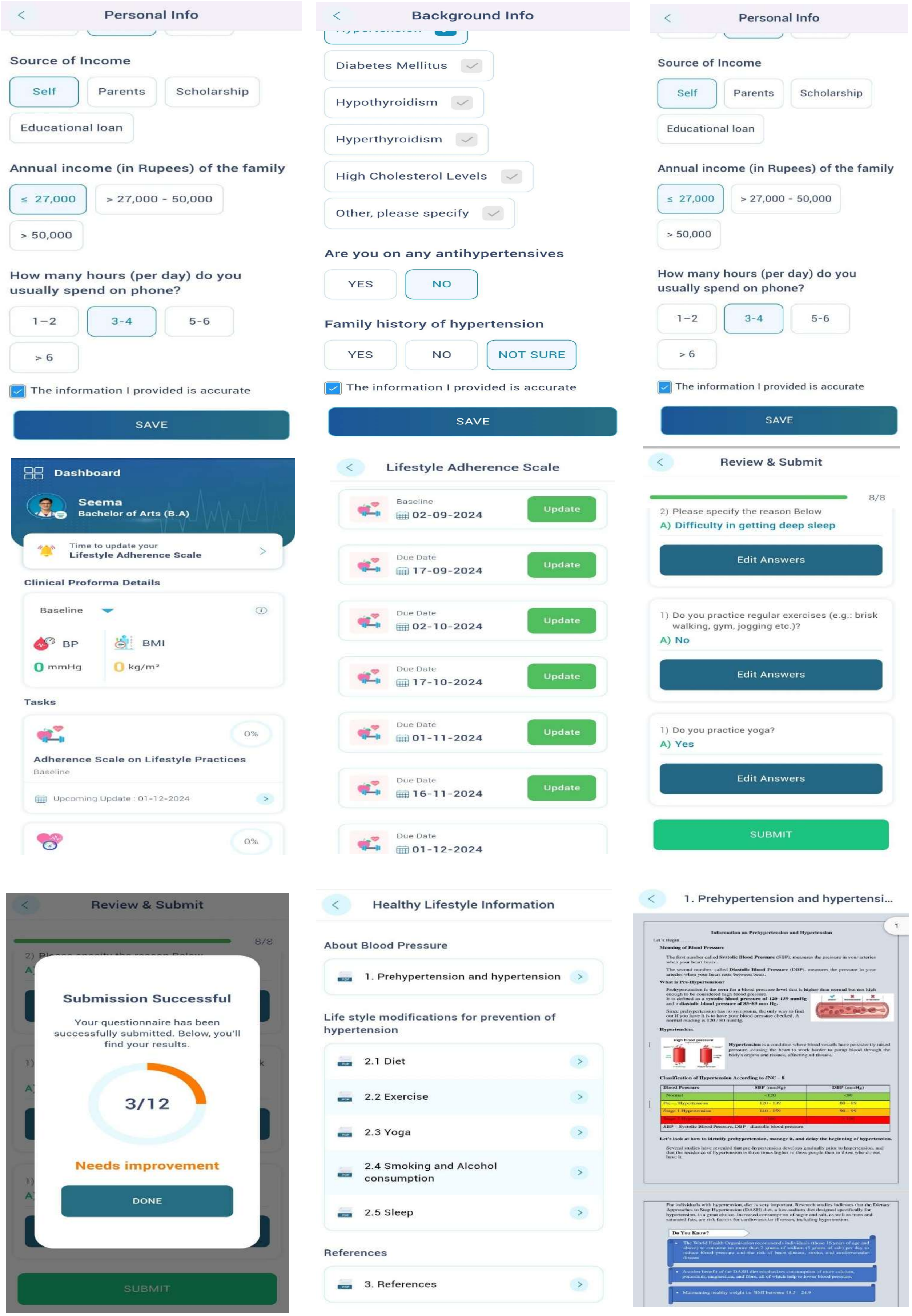

Personal Info
Source of Income
Self
Parents
Scholarship
Educational loan
Annual income (in Rupees) of the family
≤ 27,000
> 27,000 - 50,000
> 50,000
How many hours (per day) do you usually spend on phone?
1–2
3-4
5-6
> 6
The information I provided is accurate
SAVE
Background Info
Diabetes Mellitus
Hypothyroidism
Hyperthyroidism
High Cholesterol Levels
Other, please specify
Are you on any antihypertensives
YES
NO
Family history of hypertension
YES
NO
NOT SURE
The information I provided is accurate
SAVE
Personal Info
Source of Income
Self
Parents
Scholarship
Educational loan
Annual income (in Rupees) of the family
≤ 27,000
> 27,000 - 50,000
> 50,000
How many hours (per day) do you usually spend on phone?
1–2
3-4
5-6
> 6
The information I provided is accurate
SAVE
Dashboard
Seema
Bachelor of Arts (B.A)
Time to update your
Lifestyle Adherence Scale
Clinical Proforma Details
Baseline
BP
BMI
0 mmHg
0 kg/m²
Tasks
0%
Adherence Scale on Lifestyle Practices
Baseline
Upcoming Update : 01-12-2024
0%
Lifestyle Adherence Scale
Baseline
02-09-2024
Update
Due Date
17-09-2024
Update
Due Date
02-10-2024
Update
Due Date
17-10-2024
Update
Due Date
01-11-2024
Update
Due Date
16-11-2024
Update
Due Date
01-12-2024
Review & Submit
8/8
2) Please specify the reason Below
A) Difficulty in getting deep sleep
Edit Answers
1) Do you practice regular exercises (e.g.: brisk walking, gym, jogging etc.)?
A) No
Edit Answers
1) Do you practice yoga?
A) Yes
Edit Answers
SUBMIT
Review & Submit
8/8
Submission Successful
Your questionnaire has been successfully submitted. Below, you'll find your results.
3/12
Needs improvement
DONE
SUBMIT
Healthy Lifestyle Information
About Blood Pressure
1. Prehypertension and hypertension
Life style modifications for prevention of hypertension
2.1 Diet
2.2 Exercise
2.3 Yoga
2.4 Smoking and Alcohol consumption
2.5 Sleep
References
3. References
1. Prehypertension and hypertensi...
Information on Prehypertension and Hypertension
Meaning of Blood Pressure
What is Pre-Hypertension?
Hypertension:
Classification of Hypertension According to JNC - 8
Blood Pressure
SBP (mmHg)
DBP (mmHg)
Do You Know?

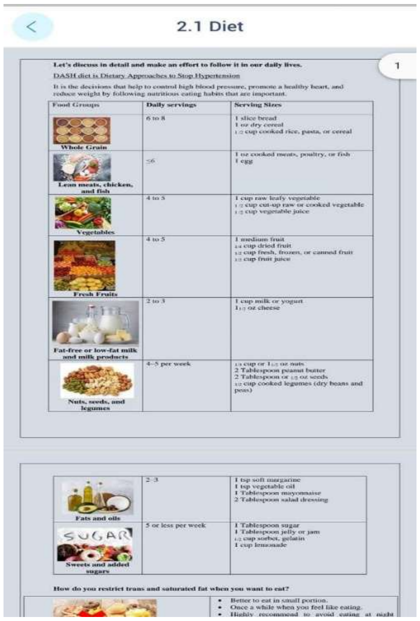

2.1 Diet

Let's discuss in detail and make an effort to follow it in our daily lives.

DASH diet is Dietary Approaches to Stop Hypertension

It is the decisions that help to control high blood pressure, promote a healthy heart, and reduce weight by following nutritious eating habits that are important.

Food Groups	Daily servings	Serving Sizes
Whole Grain	6 to 8	1 slice bread<br>1 oz dry cereal<br>1/2 cup cooked rice, pasta, or cereal
Lean meats, chicken, and fish	≤6	1 oz cooked meats, poultry, or fish<br>1 egg
Vegetables	4 to 5	1 cup raw leafy vegetable<br>1/2 cup cut-up raw or cooked vegetable<br>1/2 cup vegetable juice
Fresh Fruits	4 to 5	1 medium fruit<br>1/4 cup dried fruit<br>1/2 cup fresh, frozen, or canned fruit<br>1/2 cup fruit juice
Fat-free or low-fat milk and milk products	2 to 3	1 cup milk or yogurt<br>1 1/2 oz cheese
Nuts, seeds, and legumes	4–5 per week	1/3 cup or 1 1/2 oz nuts<br>2 Tablespoon peanut butter<br>2 Tablespoon or 1/2 oz seeds<br>1/2 cup cooked legumes (dry beans and peas)
Fats and oils	2-3	1 tsp soft margarine<br>1 tsp vegetable oil<br>1 Tablespoon mayonnaise<br>2 Tablespoon salad dressing
Sweets and added sugars	5 or less per week	1 Tablespoon sugar<br>1 Tablespoon jelly or jam<br>1/2 cup sorbet, gelatin<br>1 cup lemonade

How do you restrict trans and saturated fat when you want to eat?

- Better to eat in small portion.
- Once a while when you feel like eating.
- Highly recommend to avoid eating at night

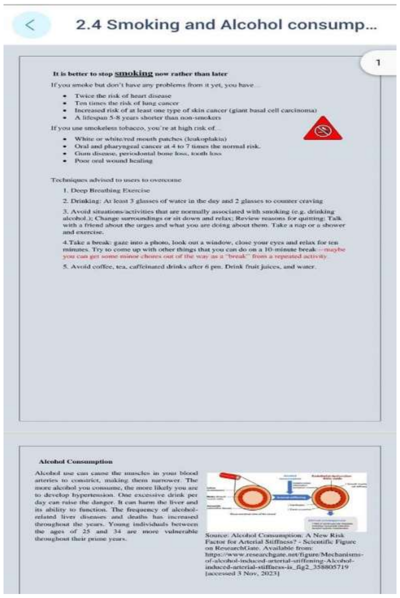

2.4 Smoking and Alcohol consump...

It is better to stop **smoking** now rather than later

If you smoke but don't have any problems from it yet, you have...

- Twice the risk of heart disease
- Ten times the risk of lung cancer
- Increased risk of at least one type of skin cancer (giant basal cell carcinoma)
- A lifespan 5-8 years shorter than non-smokers

If you use smokeless tobacco, you're at high risk of...

- White or white/red mouth patches (leukoplakia)
- Oral and pharyngeal cancer at 4 to 7 times the normal risk.
- Gum disease, periodontal bone loss, tooth loss
- Poor oral wound healing

Techniques advised to users to overcome

1. Deep Breathing Exercise
2. Drinking: At least 3 glasses of water in the day and 2 glasses to counter craving
3. Avoid situations/activities that are normally associated with smoking (e.g. drinking alcohol.); Change surroundings or sit down and relax; Review reasons for quitting; Talk with a friend about the urges and what you are doing about them. Take a nap or a shower and exercise.
4. Take a break: gaze into a photo, look out a window, close your eyes and relax for ten minutes. Try to come up with other things that you can do on a 10-minute break —maybe you can get some minor chores out of the way as a "break" from a repeated activity.
5. Avoid coffee, tea, caffeinated drinks after 6 pm. Drink fruit juices, and water.

Alcohol Consumption

Alcohol use can cause the muscles in your blood arteries to constrict, making them narrower. The more alcohol you consume, the more likely you are to develop hypertension. One excessive drink per day can raise the danger. It can harm the liver and its ability to function. The frequency of alcohol-related liver diseases and deaths has increased throughout the years. Young individuals between the ages of 25 and 34 are more vulnerable throughout their prime years.

Source: Alcohol Consumption: A New Risk Factor for Arterial Stiffness? - Scientific Figure on ResearchGate. Available from: https://www.researchgate.net/figure/Mechanisms-of-alcohol-induced-arterial-stiffening-Alcohol-induced-arterial-stiffness-is_fig2_358805719 [accessed 3 Nov, 2023]

Admin Panel

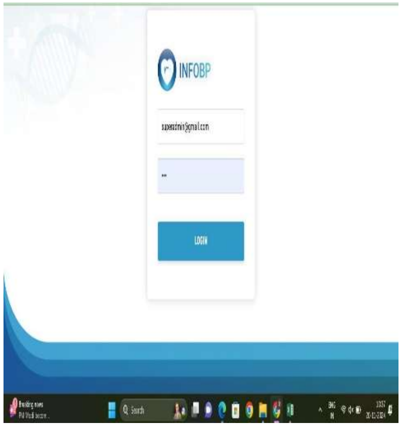

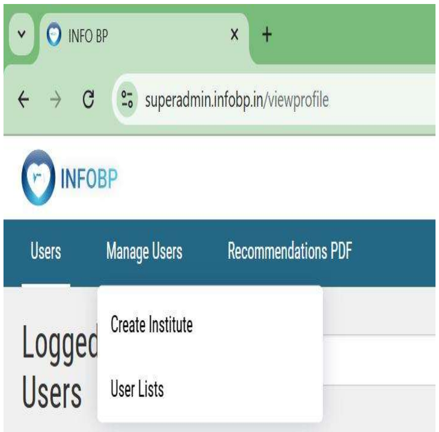

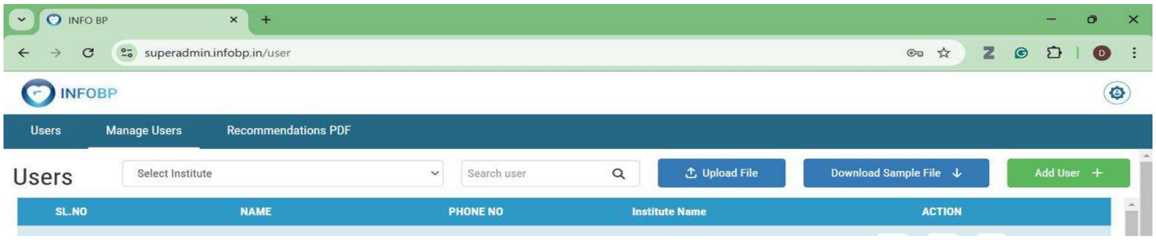

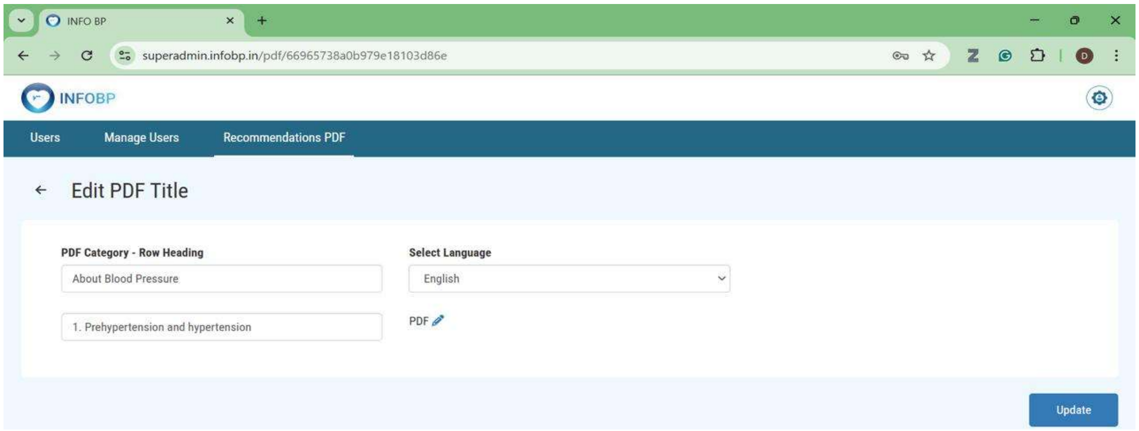

Supplement: Multimedia Appendix 2 [file resprot_v14i1e67216_app2.pdf]
