# Supplementary material for: Effectiveness of an mHealth Program on Reducing Blood Pressure Among Young Adults With Prehypertension: Protocol of a Pragmatic Cluster Randomized Controlled Trial
Source: JMIR Res Protoc. 2025 Aug 7;14:e67216. doi: 10.2196/67216 (PMC12371297; doi:10.2196/67216)
Supplement: Multimedia Appendix 5 [file resprot_v14i1e67216_app5.docx]

| **SN** | **Engagement** | **Functionality** | **Aesthetics** | **Information** | **MARS Mean** | **Subjective** | **Perceived Impact** | **General description** |
| --- | --- | --- | --- | --- | --- | --- | --- | --- |
| 1 | 4.2 | 4.5 | 5 | 3.29 | 4.25 | 3.00 | 5.00 | No comments |
| 2 | 3.8 | 2.75 | 4 | 3 | 3.39 | 2.25 | 3.50 | No comments |
| 3 | 3.2 | 4.75 | 4.67 | 4 | 4.15 | 3.50 | 4.00 | No comments |
| 4 | 3.6 | 4 | 3.67 | 3.5 | 3.69 | 3.50 | 4.50 | No comments |
| 5 | 2.8 | 3.5 | 3.33 | 3.75 | 3.35 | 2.75 | 3.83 | No comments |
| 6 | 4.2 | 3.5 | 3.5 | 4.6 | 3.95 | 3.75 | 5.00 | No comments |
| 7 | 3.2 | 1.5 | 3.67 | 3.5 | 2.97 | 3.50 | 5.00 | This app is very helpful |
| 8 | 3.6 | 2.5 | 2.67 | 3.25 | 3.00 | 3.25 | 3.17 | Very Good, useful and fantastic |
| 9 | 3.6 | 5 | 4 | 3.25 | 3.96 | 4.00 | 5.00 | This app is very useful in students’ life |
| 10 | 4 | 4.25 | 4.67 | 4 | 4.23 | 3.75 | 4.33 | helpful to students |
| 11 | 3.6 | 3.25 | 4.33 | 1.71 | 3.22 | 2.75 | 3.67 | Informative, change the attitude towards improving healthy behaviours, increase knowledge |
| 12 | 4.2 | 4.25 | 4.33 | 4.75 | 4.38 | 3.75 | 3.67 | Gain knowledge regarding the health |
| Overall | 3.67 | 3.65 | 3.99 | 3.55 | 3.71 | 3.31 | 4.22 |  |

Response of the Participants obtained using Mobile Application Rating Scale (MARS) (N=12)
